# Absorption and Extinction Cross Sections and Photon Streamlines in the Optical Near-field

**DOI:** 10.1038/s41598-017-15528-w

**Published:** 2017-11-13

**Authors:** Moritz Striebel, Jӧrg Wrachtrup, Ilja Gerhardt

**Affiliations:** 1 Institute of Physics, University of Stuttgart and Center for Integrated Quantum Science and Technology (IQST), Pfaffenwaldring 57, D-70569 Stuttgart, Germany; 2 Max Planck Institute for Solid State Research, Heisenbergstraße 1, D-70569 Stuttgart, Germany

## Abstract

The optical interaction of light and matter is modeled as an oscillating dipole in a plane wave electromagnetic field. We analyze absorption, scattering and extinction for this system by the energy flow, visualized as streamlines of the Poynting vector. Depending on the dissipative damping of the oscillator, a part of the streamlines ends up in the dipole. Based on a graphical investigation of the streamlines, this represents the absorption cross section, and forms a far-field absorption aperture. In the near-field of the oscillator, a modification of the aperture is observed. As in the case for a linear dipole, we model the energy flow and derive the effective absorption apertures for an oscillator with a circular dipole characteristics – such as an atom in free space.

## Introduction

One of the most fundamental processes which involves the interaction of light and matter is the attenuation of light by a nanoscopic emitter. Its fundamental limit is defined by the interaction of a beam and a point-like dipole. The introduced attenuation of light is observed since ancient times. Today’s nano-optic technologies have enabled controlled experiments with single photons^1^ and single nano-scale emitters^2^. The theoretical description of this optical interaction does not only address very fundamental questions, it might lead to an increased efficiency in light-matter interaction. While much attention has been paid to engineering optimal light extraction strategies^3,4^ – so that a single molecule or a nano-crystal can be detected by fluorescence with a good signal to noise ratio – relatively little has been paid to achieving the most efficient excitation. For optical protocols, such as the quantum phase gate^5^, or coherent microscopy schemes^6^, an efficient excitation and extraction of light – ideally a single photon – to and from a single emitter is desirable.

Light attenuation by a single molecules^2,7,8^, quantum dots^9^, atoms^10–13^, and recently NV-centers^14^ has been investigated. The initial solid state experiments were initially conducted under cryogenic conditions^2,8^ to achieve the required signal-to-noise ratio. Only balanced detection^15^ made it possible to extend these fundamental studies to room temperature^16,17^. Motivated by the experimental realization of these fundamental processes, numerous theoretical approaches were reinvented^18,19^, often founded on electrodynamic calculations from the 1960s when the field of antenna theory was very active. One central question was the maximum amount of extinction in a real optical focus^18^. Others describe the energy relations in the context of the optical theorem and in the near-field^20^. The energy flow itself, depicted as streamlines of the Poynting vector, introduced a vivid insight in this fundamental problem already decades ago^21,22^. Today, we see the most fundamental approaches are presently getting unified between classical antenna theory, quantum technologies and nano-optics.

Much of the literature on the interaction of light and point dipoles uses *cross sections* to quantify the distinction between the total amount of “incident radiation” and light which is then *either* scattered (extinction cross section) *or* absorbed by the dipole (absorption cross section). The optical far-field is the only regime where the cross sections are strictly defined. Here, we nevertheless approach the emitter and observe the non-trivial behavior of the cross section concept. The extinction is also famously related to the *optical theorem*, that explains how the total amount of extinction manifests itself through the reduction of power detected in the forward scattering direction as compared to when no dipole is present. These concepts relate directly to a plane wave excitation of the dipole, and almost always assume a real, scalar dipole transition moment – which corresponds to a symmetrically excited linear or isotropic dipole.

Many modern investigations involve highly-focused or sub-wavelength apertured excitation and/or detection geometries, and dipoles with complex and/or an-isotropic transition moments. The relevance of cross sections and the extinction theorem to non-plane-wave excitation conditions is often tenuous at best, and geometry-specific models must be developed to properly account for the important role played by the near-field terms in the fields scattered by the dipole. In some cases the impact of the scattering geometry can lead to behavior that seemingly contradicts the results and intuition associated with the plane-wave results.

In this paper we review the fundamental basics of coherent light-matter interaction and absorption of a single emitter. The relevant cross sections of absorption, extinction and scattering are analytically derived from first principles for plane wave excitation. Everything is applied to the mathematical description of the energy flow in the proximity of a dipolar emitter. While this was discussed already in the past decade, we address shortcomings and present novel findings. The cross sections are calculated, and the analytic result and their derivation by following the energy flow are compared. In the close proximity of the dipole the total field is altered by the presence of the emitter. This change also alters the effective cross sections. The (optical) near-field cross sections become more and more a dipolar shape. Whereas the dipolar emitter was assumed as a linear, Hertzian, dipole in many papers, we see the experimental efforts extended to single atoms^11,13^, which usually exhibit a circular dipole. Therefore, we further adapt the underlying formulation to the case of a circular dipole.

The paper is organized as follows: In section 2 the basic effect of light extinction and absorption is reviewed. It starts with a perfect dipole, introduces the polarizability and the loss-channels. Then, a visual representation of the cross sections is presented. Section 3 introduces the use of apertures which illustrate the mentioned cross-sections. Section 4 introduces the theory on a circular dipole emitter, such as it is represented by an atom in free space.

## Absorption, Scattering and Extinction

### A perfect dipole and a plane wave

**Figure 1:**
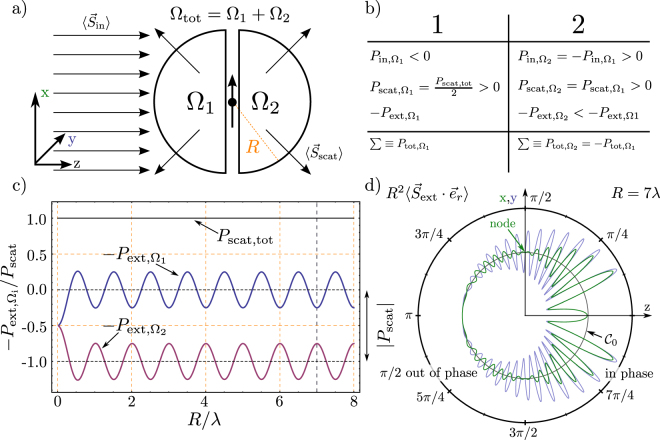
(**a**) An electromagnetic wave is incident on a dipole, which scatters all energy without further loss. An integrating sphere is divided into a forward (${{\rm{\Omega }}}_{2}$) and a backward (${{\rm{\Omega }}}_{1}$) direction (**b**) The different energy contributions. (**c**) Forward (${P}_{{\mathrm{ext},{\rm{\Omega }}}_{{\rm{2}}}}$, red) and backward (${P}_{{\mathrm{ext},{\rm{\Omega }}}_{{\rm{1}}}}$, blue) component dependent on the radius of the sphere as well as ${P}_{\mathrm{scat},\mathrm{tot}}$. The situation corresponding to (**d**) is marked with a gray dashed line. The hole effect of extinction occurs in forward direction and is equal to the scattered amount of power as required by energy conservation and we have $Extinction=Scattering$ (no absorption). The oscillation in the extinction terms arise from integration effects of the plane wave over a spherical surface d) Solutions for ${{R}^{2}\langle {\overrightarrow{S}}_{{\rm{ext}}}\cdot {\overrightarrow{e}}_{{\rm{r}}}\rangle |}_{yz}$ (blue) and ${{R}^{2}\langle {\overrightarrow{S}}_{{\rm{ext}}}\cdot {\overrightarrow{e}}_{{\rm{r}}}\rangle |}_{xz}$ (green). The quantity ${R}^{2}\langle {\overrightarrow{S}}_{{\rm{ext}}}\cdot {\overrightarrow{e}}_{{\rm{r}}}\rangle$ represents the integrator of the extinction term. The reference curve ${{\mathscr{C}}}_{0}$ defines zero. The magnitude of the solutions is plotted against the reference curve.

We approach the relevant math with an intuitive picture of the fundamental process of light-matter interaction. To that end we study the most basic case, given by the interaction of an incident plane wave with a single dipole. A single dipole emitter is assumed in free space at the origin of a coordinate system (Fig. 1a). An incident plane wave, which propagates from $-z$ to $+z$ excites the emitter. This field is expressed as

1a $${\overrightarrow{E}}_{{\rm{in}}}={E}_{{\rm{in}}}^{{\rm{0}}}\hat{\varepsilon }\exp ({\rm{i}}kz-{\rm{i}}\omega {\rm{t}})$$

1b $${\overrightarrow{B}}_{{\rm{in}}}=\frac{1}{c}\hat{k}\times {\overrightarrow{E}}_{{\rm{in}}},$$

where $\hat{\varepsilon }$ is the Jones vector of the incident wave.

Let us consider an electrically polarizable system – such as a dipole – in vacuum. This can be excited by a incident electric field ${\overrightarrow{E}}_{{\rm{in}}}\mathrm{(0)}$ at the location of the dipole, $\overrightarrow{d}$, the response of the system due to the incident field is given by

2 $$\overrightarrow{d}=\alpha {\overrightarrow{E}}_{{\rm{in}}}\mathrm{(0).}$$

Here, $\alpha$ is the electric polarizability of the dipole. The system is assumed to exhibit a parallel response due to the incident field, and treated as a point-like dipole, i.e. infinitely small. In general, the response of the system is not necessarily parallel to the incident field, and $\alpha$ has to be written as a tensor. This limitation is relaxed in the following section. As a consequence of the excitation, the dipole scatters a certain amount of power into all 4$\pi$ steradians of the environment (Fig. 1a).

In the following we calculate the time averaged energy flow into the system, to get an idea how energy is absorbed by the dipole. To do so, we calculate the time averaged Poynting vector, which is computed by (for time harmonic fields)^23^

3 $$\overrightarrow{S}=\frac{1}{2{\mu }_{0}} {\mathcal R} e\{{\overrightarrow{E}}_{{\rm{tot}}}\times {\overrightarrow{B}}_{{\rm{tot}}}^{\ast }\}.$$

Here, ${\overrightarrow{E}}_{{\rm{tot}}}$ is the total electric field calculated as a superposition of the incident field and the scattered field. ${\overrightarrow{B}}_{{\rm{tot}}}$ denotes the corresponding magnetic field. Since magnetic response is neglected, ${\overrightarrow{B}}_{{\rm{tot}}}={\mu }_{0}{\overrightarrow{H}}_{{\rm{tot}}}$ holds.

The scattered dipole field is expressed by^23^

4 $${\overrightarrow{E}}_{{\rm{scat}}}={d}_{0}\{\frac{{k}^{2}}{r}((\hat{r}\times \hat{d})\times \hat{r})+[\mathrm{3(}\hat{r}(\hat{r}\cdot \hat{d}-\hat{d}))](\frac{1}{{r}^{3}}-\frac{{\rm{i}}k}{{r}^{2}})\}\exp \,({\rm{i}}kr)$$

4b $${\overrightarrow{B}}_{{\rm{scat}}}={k}^{2}{d}_{0}(\hat{r}\times \hat{d})(1-\frac{1}{{\rm{i}}kr})\frac{\exp ({\rm{i}}kr)}{r},$$

where $\hat{d}$ is the unit vector in the direction of the induced dipole moment and ${d}_{0}$ is the magnitude of the dipole moment ($\overrightarrow{d}={d}_{0}\cdot \hat{d}$). Since we said that the dipole moment is parallel to the incident wave polarization, $\hat{\varepsilon }=\hat{d}$ is valid. The total energy flow is written as

$$\overrightarrow{S}=\mathop{\underbrace{\frac{1}{2{\mu }_{0}} {\mathcal R} {e}\{{\overrightarrow{E}}_{{\rm{in}}}\times {\overrightarrow{B}}_{{\rm{in}}}^{\ast }\}}}\limits_{\langle {\overrightarrow{S}}_{{\rm{in}}}\rangle }+\mathop{\underbrace{\frac{1}{2{\mu }_{0}} {\mathcal R} {e}\{{\overrightarrow{E}}_{{\rm{in}}}\times {\overrightarrow{B}}_{{\rm{scat}}}^{\ast }+{\overrightarrow{E}}_{{\rm{scat}}}\times {\overrightarrow{B}}_{{\rm{in}}}^{\ast }\}}}\limits_{\langle {\overrightarrow{S}}_{{\rm{ext}}}\rangle }+\mathop{\underbrace{\frac{1}{2{\mu }_{0}} {\mathcal R} {e}\{{\overrightarrow{E}}_{{\rm{scat}}}\times {\overrightarrow{B}}_{{\rm{scat}}}^{\ast }\}}}\limits_{\langle {\overrightarrow{S}}_{{\rm{scat}}}\rangle }\mathrm{.}$$

We now analyze the energy distribution in the case of a dipole that dissipates energy only via elastic scattering of the incident wave. Therefore, the space is separated into two half spheres, one in the forward direction (${{\rm{\Omega }}}_{{\rm{2}}}$) and one in the backward direction (${{\rm{\Omega }}}_{{\rm{1}}}$). Afterwards, the transmitted energy is analyzed. The different contributions and their signs, i.e. if they are directed inwards to (−) or outwards from the sphere (+), are summarized in the table Fig. 1b. As expected, we find that the sum of the energy contributions which travels through the sphere in the forward direction and in the backward direction yield to the same amount with different signs so that their total sum is zero. This is required by energy conservation. The extinction terms are here listed as negative, since the extinguished power is commonly defined by

5 $${P}_{{\rm{ext}}}=-{\int }_{{{\rm{\Omega }}}_{{\rm{tot}}}}{\overrightarrow{S}}_{{\rm{ext}}}\,d{\rm{\Omega }}\mathrm{.}$$

**Figure 2:**
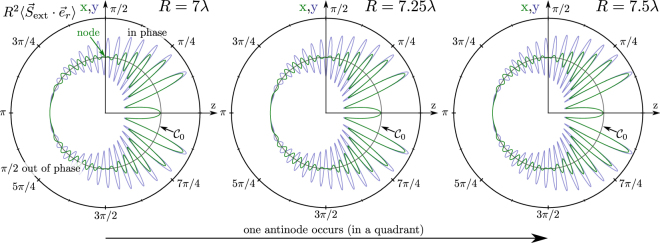
Solutions for ${{R}^{2}\langle {\overrightarrow{S}}_{{\rm{ext}}}\cdot {\overrightarrow{e}}_{{\rm{r}}}\rangle |}_{yz}$ (blue) and ${{R}^{2}\langle {\overrightarrow{S}}_{{\rm{ext}}}\cdot {\overrightarrow{e}}_{{\rm{r}}}\rangle |}_{xz}$ (green) for values of $R$ chosen in distances of $\lambda \mathrm{/4}$. One anti-node occurs within half a wavelength. This periodic behavior has been described in context of Fig. 1c. The plots shown here are closely related to the results of Ref.^24^.

The incident power (${P}_{{\rm{in}}}$) is symmetric and has the same amount in the forward and the backward direction with a different sign. The scattered power (${P}_{{\rm{scat}}}$) is symmetric and amounts to the same value backwards and forwards (dipole radiation). Interestingly, the extinction terms are not symmetric anymore. Whereas power in the backwards direction oscillates around zero with a distance $R$ to the emitter (blue curve), the power in the forward direction oscillates around the negative value of the total scattered power (red curve, Fig. 1c). It might be confusing that this integration is not independent of $R$ (radius of the integrating half sphere). The incident plane wave has no constant phase on the surface of a half sphere, thus the integral depends on the radius and therefore on how many wave peaks and valleys are collected. Both extinction terms together add up to the amount of the scattered power in all 4$\pi$ steradians. This becomes clear when the energy conservation is considered. The oscillation amplitude amounts to half of the power of the scattered power. This is a result of a spurious phase difference in both planes, which are displayed in Figs 1 and 2.

**Figure 3:**
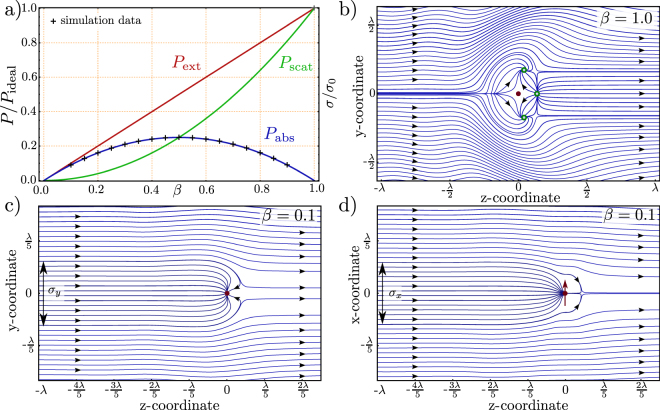
(**a**) Ratio between extinction, scattering and absorption, dependent on the loss parameter $\beta$. At *β* = 0.5 the absorption, which is an energy transfer to other than coherent re-radiation, displays a maximum. A broadband lossy particle, with *β* = 0, which has only loss, does not absorb or scatter any light. This is equivalent to a polarizability *α* = 0. A perfect scatterer, with *β* = 1, does not absorb any light. ${P}_{{\rm{ideal}}}$ corresponds to the case *β* = 1 and amounts to $\mathrm{(3}c)/\mathrm{(4}{k}^{2}){|{E}_{{\rm{in}}}^{0}|}^{2}$. This is equivalent to the consideration of the cross sections in Eqn. 21. Due to energy conservation the whole extracted energy is re-radiated. (**b**) Streamlines of the time averaged energy flow of a perfect scatterer in a plane wave. Energy used for its excitation is re-radiated, such that on average no energy is absorbed i.e. in the time averaged picture we do not see streamlines which end up in the dipole. (**c**) Streamlines for a lossy system, the loss-parameter *β* is set to 0.1, the emitter is an effective sink of energy. Therefore, some streamlines end up in the emitter. Here, the $yz$-plane is shown. (**d**) same physical situation pictured in the $xz$-plane. The extensions of the cross-section ${\sigma }_{x}$ and ${\sigma }_{{\rm{y}}}$ are not necessarily the same (see also Fig. 5b).

When the oscillations which are caused by the incident wave are neglected, it becomes clear that extinction occurs only in the forward direction and the amount adds up to ${P}_{{\rm{scat}}}$ (c.f. Fig. 3c). This corresponds to the definition of extinction as scattering plus absorption^20,24^, since we have assumed in this case that there is no absorption (such as a transfer to heat). Figure 1d shows the extinction part of the Poynting vector in the $xz$- as well as in the $yz$-plane. We have a small constant effect on the $z$-axis in the forward direction, independent of the radius (c.f. Fig. 2). This is observed as a small deviation of the reference level ${{\mathscr{C}}}_{0}$ and ${R}^{2}\langle {\overrightarrow{S}}_{{\rm{ext}}}\cdot {\overrightarrow{e}}_{{\rm{r}}}\rangle$. This effect occurs, since the phase relation between the scattered and incident wave is constant on the $z$-axis. If the behavior of this plot is studied dependent on the radius ($R$), one anti-node occurs within a half wavelength as shown in Fig. 2. Moreover, there is a phase shift of $\pi \mathrm{/2}$ between the ${R}^{2}{\langle {\overrightarrow{S}}_{{\rm{ext}}}\cdot {\overrightarrow{e}}_{{\rm{r}}}\rangle |}_{yz}$ (blue) and the ${R}^{2}\langle {{\overrightarrow{S}}_{{\rm{ext}}}\cdot {\overrightarrow{e}}_{{\rm{r}}}\rangle |}_{xz}$ (green) solutions on the incident half sphere (${{\rm{\Omega }}}_{1}$). The sum of the two parts in the forward and the backward direction always yields to zero as expected by energy conservation.

We now compare the situation investigated here with Ref.^18^, where the reflection from a single (e.g. molecular) dipole is observed. In that case, the pattern of forward scattered radiation from the dipole closely matches the forward propagating Gaussian excitation beam, but its phase is shifted by $\pi \mathrm{/2}$ due to the Gouy phase shift which spans over the focus, and the $\pi /2$ phase lag of the dipole field at resonance. Thus the total forward propagating beam is fully cancelled, and there is only a (backward) reflected beam that conserves the power flow. In the plane wave situation, no Gouy phase shift is present in the driving field. Subsequently, the patttern of forward scattered dipole radiation is completely different from the plane wave, so the “extinction” in the forward direction is limited in opposite as in the case of the focussed Gaussian.

### The polarizability, α

The model is completed by a definition of the polarizability $\alpha$. We assume the emitter is coupled to both the vacuum (which causes scattering and radiation reaction, but no extraction of power from the electromagnetic field), and to other bath(s) that can additionally dephase the dipole response. Thereby, the emitter extracts power from the electromagnetic field distribution (i.e. it generates heat). This diminishes the re-radiation of energy as the oscillator becomes a sink for the energy.

If a Lorentzian oscillator is considered, the problem can be described by the following equation of motion^23^

6 $$m(\frac{{{\rm{d}}}^{2}}{{{\rm{dt}}}^{2}}\overrightarrow{r}-\tau \frac{{{\rm{d}}}^{3}}{{{\rm{dt}}}^{3}}\overrightarrow{r}+{\Gamma }_{{\rm{nrad}}}\frac{{\rm{d}}}{{\rm{dt}}}\overrightarrow{r}+{\omega }_{0}\overrightarrow{r})=e{{\rm{E}}}_{0}\hat{\varepsilon }{{\rm{e}}}^{-{\rm{i}}\omega t}$$

where $m$ is the free electron mass and

7 $$\tau =\frac{1}{4\pi {\varepsilon }_{0}}\,\frac{2{e}^{2}}{3m{c}^{3}}.$$

${\Gamma }_{{\rm{nrad}}}$ represents the rate of dissipative loss to by the dipole. When the equation of motion is solved, it yields a expression for the inverse polarizability and uses $\overrightarrow{d}(t)=\alpha {\overrightarrow{E}}_{in}^{o}=e\overrightarrow{r}(t)$[^25^].

8 $$\frac{1}{\alpha }=\frac{m}{{e}^{2}}({\omega }_{0}^{2}-{\omega }^{2}-{\rm{i}}\omega {\Gamma }_{{\rm{t}}})$$

where

9 $${\Gamma }_{{\rm{t}}}(\omega )={\Gamma }_{{\rm{nrad}}}+{(\frac{\omega }{{\omega }_{0}})}^{2}\Gamma \,,$$

and where and $\Gamma ={\omega }_{0}^{2}\tau$ is the damping due to radiation reaction i.e. scattering.

In the remainder of the manuscript we only consider the system response when excited on resonance. For a treatment for the off-resonant case we refer to Ref.^26^. This is conveniently expressed in terms of the response function in the absence of dissipative damping as,

10 $${\alpha }_{res}=\frac{{\alpha }_{0}}{1+m{\omega }_{0}{\Gamma }_{{\rm{nrad}}}6{\pi }{\varepsilon }_{0}/({e}^{2}{k}^{3})},$$

where

11 $${\alpha }_{0}=\frac{6\pi {\varepsilon }_{0}}{{k}^{3}}{\rm{i}}$$

If no dissipation occurs (i.e. $\alpha$ is purely real), then the amount of extinction is equivalent to scattering. We consider the time averaged power extracted by the dipole by^26^

12 $${P}_{{\rm{ext}}}=-\frac{\omega }{2} {\mathcal R} e\{{\int }_{V}{\vec{J}}^{\ast }{\vec{E}}_{{\rm{in}}}\,{\rm{d}}V\}=\frac{\omega }{2}\Im {\rm{m}}\{\alpha \}{|{\vec{E}}_{{\rm{in}}}^{0}|}^{2}\,\mathrm{.}$$

Here, $\overrightarrow{J}$ is the electric current density (a harmonic time dependence $\exp (-{\rm{i}}\omega t)$ is used). For Eqn. 12 it is essential that the electric field is uniform and time harmonic over the volume, $V$, of the system (which is a semi-classical approach). The time averaged energy of the incident wave is

13 $${P}_{{\rm{in}}}=\frac{c{\varepsilon }_{0}}{2}{|{E}_{{\rm{in}}}^{0}|}^{2}\mathrm{.}$$

An oscillating dipole radiates power and the the scattered energy is given by^23^

14 $${P}_{{\rm{scat}}}=\frac{c{k}^{4}}{12\pi {\varepsilon }_{0}}|\alpha {|}^{2}{|{E}_{{\rm{in}}}^{0}|}^{2}\,=\frac{{k}^{4}}{6\pi {\varepsilon }_{0}}|\alpha {|}^{2}{P}_{{\rm{in}}}\mathrm{.}$$

If no energy is transferred into other channels (e.g. heat) the amount of extracted and scattered energy is equal. This implies that the absorption is zero. In the time averaged case no energy flows into the dipole. All energy which is transferred to the emitter first excites the emitter and is then re-radiated. In the following, the polarizability is defined to account for further loss channels.

### Introduction of a loss channel, *β*

The introduction of $\alpha$ as outlined above allows the introduction of a loss parameter, $\beta$, which is sometimes also introduced as a “single-scattering albedo”,

15 $${\ss}=\frac{{a}_{res}}{{a}_{0}}=\frac{1}{1+m{?}_{0}{G}_{{\rm{nrad}}}6p{e}_{0}/({e}^{2}{k}^{3})}\quad \quad {\rm{where}}\quad \quad 0={\ss}=1.$$

The dipole moment is then written as

16 $$\overrightarrow{d}=\alpha {\overrightarrow{E}}_{{\rm{in}}}^{0}={\alpha }_{0}\beta {\overrightarrow{E}}_{{\rm{in}}}^{0}\mathrm{.}$$

In this notation the factor *β* can be seen as a heuristically introduced absorption parameter, since the polarizability of the system is reduced if absorption is introduced. The different power flow parameters on resonance, are given by

17a $${{P}}_{ext}=\frac{3c{\pi }{{\varepsilon }}_{0}}{{k}^{2}}{\beta }{|{{E}}_{in}^{0}|}^{2}$$

17b $${{P}}_{{\rm{scat}}}=\frac{3c{\pi }{{\varepsilon }}_{0}}{{k}^{2}}{{\beta }}^{2}{|{E}_{in}^{0}|}^{2}$$

17c $${P}_{{\rm{abs}}}={P}_{{\rm{ext}}}-{P}_{{\rm{scat}}}=\frac{3c\pi {\varepsilon }_{0}}{{k}^{2}}(\beta -{\beta }^{2})|{E}_{{\rm{in}}}^{0}{|}^{2},$$

where the relation $k=\omega /c$ is used. The three different contributions in Eqn. 5 correspond to the different energy components given in Eqn. 17a,b,c via an integration over an imaginary sphere ${{\rm{\Omega }}}_{{\rm{tot}}}$ (c.f. Fig. 1) which incooperates the dipole.

Since $\beta$ is given by the ratio

18 $$\beta =\frac{{P}_{{\rm{scat}}}}{{P}_{{\rm{ext}}}}=\frac{{P}_{{\rm{scat}}}}{{P}_{{\rm{abs}}}+{P}_{{\rm{scat}}}}\mathrm{.}$$

We see that for $\beta  < 0.5$ the amount of absorbed power dominates the ratio, and for $\beta  > 0.5$ the amount of scattered energy dominates (c.f. Fig. 3a). Equation 18 shows the meaning of $\beta$ as the fraction of re-radiated energy to the total extracted energy. In this context it should be mentioned that G. Wrigge derives an analogous result for a two-level-system with decay channels (below saturation) in Ref.^27^, that shows the universality of this fact illustrated in Fig. 3a. It might be confusing that some textbooks (e.g.^28^) handle Eqn. 12 as the *absorbed* power. If they do so, they use a quasi-static description of the polarizability. In this case scattering by the system is neglected, and extinction and absorption become the same. It has to be mentioned that the quasi static polarizability conflicts with the optical theorem, but Eqn. 8 provides a solution to this dilemma.

To visualize the energy flow we compute the time averaged Poynting vector and solve the differential Eqn. 23 numerically to get its streamlines.

19 $$\frac{{{\rm{dr}}}_{i}}{{\rm{d}}s}={S}_{i}(x(s),y(s),z(s))\quad \quad {\rm{where}}\quad \quad i\hat{=}\{x,y,z\},$$

with $s$ used as a “dummy” parameter. Such a picture was derived earlier. It allows for a vivid insight on the process of light absorption^21,22^.

For the earlier discussed case, where no absorption occurs, we show the case of streamlines in Fig. 3b. All streamlines are redirected, but do not end up in the dipole at the origin. It must be noted that the streamlines leave the $xy$-plane. These points are marked with circles in the plot (green). The energy flow for the case of *β* = 0.1 is shown in Fig. 3c,d. It is nicely visible how the dipole “collects” energy from a region, which is by far larger then the geometrical spread of the dipole (a point-like dipole is considered). Moreover, it is visible that the streamlines are directed towards the dipole, even if they have already “passed” the position of the emitter. The reason for that behavior is found in the interference terms. The interfere happens in such a way that the energy flow is directed towards the dipole. For a lower *β* value, the effect on the incident plane wave is smaller, as evident on the $xy$-coordinates. With a *β* of unity, the distortion of streamlines exceeds one wavelength. This effect is diminished with a lower *β*.

Based on the Eqn. 17a,b,c we see that *β* = 1 refers to the ideal system where we have no absorption and the whole energy is re-radiated, whereas the *β* = 0 case refers to no excitation, respectively no polarizability of the system. Figure 3a shows the normalized energy contributions given in Eqn. 19b,c plotted as a function of *β*. The figure shows nicely the fact that absorption is necessarily concomitant with scattering i.e. absorption without scattering can not exist. Moreover, it occurs that the maximum value of absorption is given if *β* = 0.5, which is equivalent to an equivalent amount of scattered and absorbed energy. As shown in Ref.^26^, this is also valid for the case of out of resonance.

**Figure 4:**
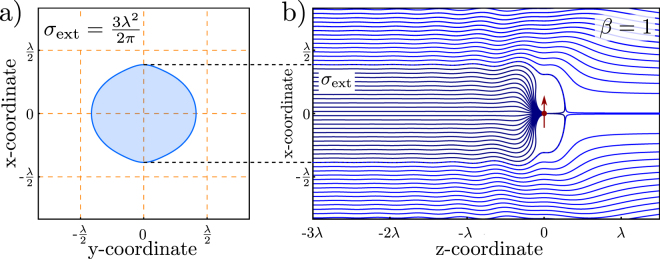
Corresponding to the *absorption* cross-section in Fig. 3b–d, the *extinction* cross section (${\sigma }_{{\rm{e}}xt}$) can be calculated. For no further loss ($\beta =1$), this amounts to $3{\lambda }^{2}\mathrm{/(2}\pi )$, as shown in the picture. To note, that this cross-section is not simply circular.

Figure 4 shows a calculation of the streamlines in the case for the pure *extinction* case. For the calculation, it simply implies to take only the first two terms of Eqn. 5, and neglect the third term. Additionally, no further losses are accounted for. This implies that the loss parameter, *β*, is unity. Of course, this implies, that we artificially disregard the amount of energy which is redistributed in all 4$\pi$ steradians – subsequently, the full incident energy is not conserved in this case. The scattering and the extinction cross-section are the same at this point, and amount to $3{\lambda }^{2}\mathrm{/(2}\pi )$, as introduced in Eqn. 21 below. In the following, we only consider the case of the *absorption* cross-section, such that a certain loss, e.g. a transfer to heat, is introduced by the emitter.

### The cross sections

Based on the Eqn. 17a,b,c it is possible to define three different on resonant cross sections. A more general description can be found using Eqn. 8

20a $${\sigma }_{{\rm{ext}}}=\frac{{P}_{{\rm{ext}}}}{{P}_{{\rm{in}}}}=\frac{6\pi }{{k}^{2}}\beta $$

20b $${\sigma }_{{\rm{scat}}}=\frac{{P}_{{\rm{scat}}}}{{P}_{{\rm{in}}}}=\frac{6\pi }{{k}^{2}}{\beta }^{2}$$

20c $${\sigma }_{{\rm{abs}}}={\sigma }_{{\rm{ext}}}-{\sigma }_{{\rm{scat}}}=\frac{6\pi }{{k}^{2}}(\beta -{\beta }^{2})\,\mathrm{.}$$

**Figure 5:**
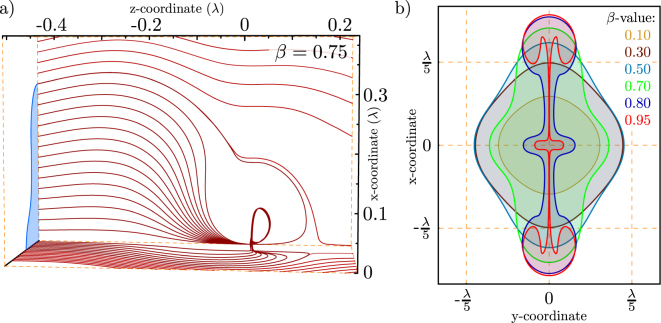
(**a**) Three dimensional representation of the energy flow into a dipole. Due to symmetry, only one quadrant is shown for $\beta =0.75$. It is visible that the streamlines leave the $yz$-plane. We determine the effective absorption aperture, by finding the boundary between the lines which end up in the dipole and the ones which are bypassing. b) the shape of this absorption aperture (equivalent to the cross section) for different values of $\beta$ far away from the emitter ($z\approx 2.4{\lambda }$). The crossed labels on the curve ${P}_{{\rm{abs}}}$ in Fig. 3a correspond to the derived areas for the different $\beta$-values in here.

As implied in Fig. 3c,d the area of streamlines which end up in the singularity (given by the dipole) suggests a relationship to the absorption cross section. For the three dimensional case, this is depicted in Fig. 5a. This definition represents a graphical association to Eqn. 20. Based on this definition the shape of the absorption cross section is derived for different loss parameters. The result is shown in Fig. 5b. To derive the absorption cross section we numerically searched for the starting point on the $x$-axis where a small deviation from the point ${\overrightarrow{r}}_{{\rm{p}}}=\{{z}_{0},\,\mathrm{0,}\,{x}_{{\rm{bound}}}\}$ yields a change of the streamlines whether they end up in the singularity or not. ${z}_{0}$ is chosen to be far away from the dipole for now, and ${x}_{{\rm{bound}}}$ denotes this boundary. Once this point is found, the boundary of the aperture can be followed by circling around the last derived point and checking the endpoint of the streamlines. Such apertures were calculated in the past^29,30^. The area of the apertures is then be derived by an interpolation of the data and the use of Greens theorem. As visible from the plot (Fig. 5b) the shape of the absorption cross section must not necessarily be round.

Moreover, it has to be noted that while the theoretical provided behavior of the absorption cross section as a function of *β* (blue curve in Fig. 3a) would intuitively yield an assumption of a shape symmetry against a value of *β* = 0.5, the shape totally differs between a pair of *β*-values symmetric to 0.5 (e.g. *β* = 0.3 and *β* = 0.7 in Fig. 5b). But the computation of the area of the absorption cross section based on the data of the simulation shows a good correspondence to the theoretical forecast (black “ + ” in Fig. 3a, i.e. the area of the pair *β* = 0.3 and *β* = 0.7 is equal). The physical interpretation of this behavior implies that the amount of energy absorbed by a pair of loss parameters symmetric to 0.5 is equal, but the way the energy “travels” into the dipole is very different.

For *β* = 1, i.e. no absorption, we find the textbook result for the extinction respectively scattering cross section of a dipole given by

21 $${\sigma }_{\mathrm{ext},\max }=\frac{6\pi }{{k}^{2}}=\frac{3{\lambda }^{2}}{2\pi }\mathrm{.}$$

Thus, the whole extinction i.e. the reduction of the incident power in the forward direction is caused by scattering. Also, other *β* values should be considered, which implies to leave the point of an ideal system without absorption. The maximum absorption cross section can be found for a loss factor of *β* = 0.5 given by

22 $${\sigma }_{\mathrm{abs},\max }=\frac{3\pi }{2{k}^{3}}=\frac{3{\lambda }^{2}}{8\pi }\mathrm{.}$$

This is again consistent with the result of Ref.^26^. So far, we did not introduce any polarization dependent response of the system, and one should note that the cross sections are indeed independent of the incident wave polarization (i.e. linear or circular). The expression for the maximum absorption cross section also holds for the off resonance case^26^. Thus, a maximum energy dissipation is generally given if ${P}_{{\rm{abs}}}={P}_{{\rm{scat}}}$. This is also visible in Fig. 3a. The results which are presented for a Hertzian dipole are equivalent to the analysis of E. Shamonina and coworkers, who evaluated the energy flow into a short antenna in Ref.^30^. The equivalence of the results is not surprising since the inverse polarizability of a short antenna is given by^26^

23 $$\frac{1}{\alpha }=\frac{{\rm{i}}\omega }{{l}^{2}}({Z}_{{\rm{inp}}}+{Z}_{{\rm{load}}})\mathrm{.}$$

Thus, the math for a short antenna is included in Eqn. 8. The parameter of interest in Ref.^30^ is ${\rm{\Delta }}$ which corresponds to *β* via $\beta =\mathrm{1/(1}+{\rm{\Delta }})$, i.e. the maximal absorption case in this nomenclature is given by ${\rm{\Delta }}=1$. The shape, we found for the absorption cross section of a Hertzian dipole is equivalent to the results of Ref.^30^.

## Apertures in the Optical Near-field

Based on the analytically expression of the cross sections given by Eqn. 20a,b,c one can easily disregard that the definition of the cross section is just strictly valid for $kr\to \infty$. A simple argument to understand this, is that the cross sections are defined via the incident power density ${P}_{{\rm{in}}}$. Since the total field is given by a superposition of the incident and the scattered field, the power density of the total field differs from the power density of the incident field. Thus, the cross sections given by Eqn. 20a,b,c define an area which yields to the total amount of extinguished, scattered and absorbed power if they are multiplied with ${P}_{{\rm{in}}}$. Hence, if the energy density of the total field changes, the area of the cross section has to change to ensure that the amount of power which is extinguished, scattered or absorbed stays the same. If the total energy density is e.g. smaller than the incident power density, the area of the cross sections has to increase, such that energy conservation is fulfilled.

**Figure 6:**
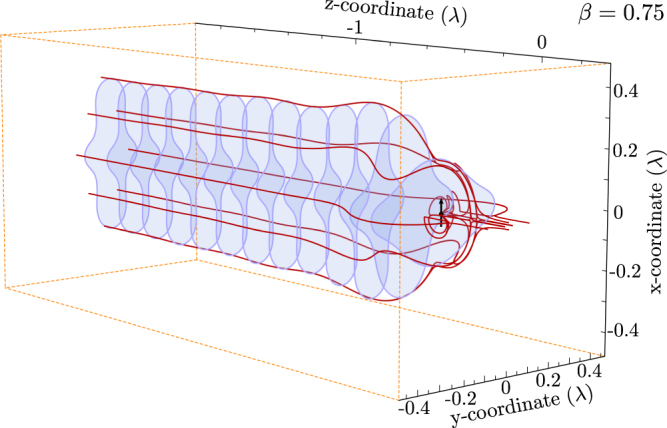
Distance dependence of the absorption cross section. Far from the emitter the streamlines in a defined region end up in the emitter. When the emitter is approached, the same amount of energy is redistributed and the effective cross section enlarges. The energy density inside the cross section is altered. A loss parameter, *β*, of 0.75 is assumed, as in Fig. 5a.

Figure 6 shows the qualitative behavior of the situation described above. The absorption aperture of a dipole with a loss factor of *β* = 0.75 is plotted along the way towards the dipole. Furthermore, some boundary solutions on and beside the aperture are shown. At the situation “far” away from the dipole, where the power density is given by the incident power density, the shape of the aperture stays constant as expected; when the emitter is approached, the shape changes.

**Figure 7:**
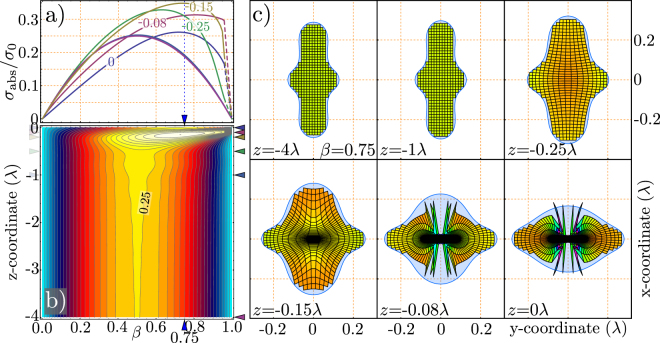
(**a**) Distance dependence of the absorption cross section. Far from the emitter, it corresponds to the theoretical description (purple, blue curve at 4 and 1 $\lambda$ distance from the dipole). The closer to the emitter the cross section is determined, the peak shifts to higher loss factors *β*. The dashed parts of the curves could not be calculated. (**b**) distance dependence in a density plot. Note that the maximum of the theoretical curve is at 0.25 ${\sigma }_{{\rm{abs}}}$. This is even not fulfilled at a distance of $z=-4\lambda$ from the dipole. (**c**) Energy density inside the absorption cross section for a loss parameter of $\beta$ = 0.75. We assume a rectangular grid, which is followed along the *z*-axis towards the emitter. It is evident, that the cross section initially widens, and it is then reshaped. As an example the energy density at $z=-0.15\lambda$ is obviously reduced, whereas the overall area is enlarged. The corresponding situation in (**a**) is marked with a blue dashed line and by triangles in (**b**).

Based on the idea Fig. 6 provides, a more detailed investigation of the aperture in the optical near-field is performed. The absorption aperture for different loss parameters is evaluated dependent on the distance to the dipole. The results are shown in Fig. 7a. The purple and the blue curve in the plot are very similar to the theoretical curve given by Eqn. 19c. As shown in the plot, the area of the aperture increases in the optical near-field (z-values are given in the plot). Moreover, it occurs, that there is a maximum value of the aperture for a certain distance in front of the dipole. A density plot, shown in Fig. 7b, shows the change in the lateral extension of the absorption cross-section. We determine the maximum at a distance of 0.15 $\lambda$ and a loss parameter *β* of 0.73. There, the effective area is 40% larger than in any far field case. Following these arguments, this directly yields a change of the total power density. As an example, the situation for a loss parameter of *β* = 0.75 is illustrated in Fig. 7c. The density of the energy flow is visualized as a grid of streamlines which are followed on their way towards the dipole. The grid is color-coded which corresponds to the energy density of the total field at the position. For the situation in the $xy$-plane at $z=-4\lambda$ the energy density appears uniform. For the corresponding situation in Fig. 7a we realize that the area nearly corresponds to the theoretical expectation in the far-field. For the situation where a maximum in the aperture area occurs ($z=-0.15$ c.f. Fig. 7a) the power density is reduced. Thus, the simulation is conformant to the previous argument.

Figure 7c shows, that the power density does not change homogeneously when the emitter is approached. Close to the emitter, the shape of the grid adapts the shape of the dipole radiation pattern (donut-shape). This indicates, that the energy flow in the near-field is dominated by the energy flow of the dipole itself. This corresponds to the result of G. Zumofen and coworkers^18^, when they describe that the dipolar component of an incident focused wave can be perfectly reflected by a single dipole. In our description, the energy flow in close proximity to the dipole seems to match such a dipolar pattern.

## Circular Dipole

If one considers an atom as a single emitter it is known from atomic physics that linear as well as circular polarized excitation is of interest. Based on the math introduced in section 2 it is simple to formulate the problem for a circular excitation. With an incident wave given by

24 $${\overrightarrow{E}}_{{\rm{in}}}={E}_{{\rm{in}}}^{0}\hat{\varepsilon }\exp ({\rm{i}}kz-{\rm{i}}\omega {\rm{t}}),$$

where $\hat{\varepsilon }\mathrm{=1/}\sqrt{2}{\mathrm{(1,}{\rm{i}},\mathrm{0)}}^{{\rm{T}}}$ corresponds to the Jones vector of a circular polarized wave. This yields to a dipole moment (on resonance)

25 $$\mathop{d}\limits^{?}={a}_{0}{\ss}\hat{e}\exp (-{\rm{i}}?t)=\frac{3}{2\sqrt{2}{k}^{3}}\beta {({\rm{i}},-\mathrm{1,}\mathrm{0)}}^{{\rm{T}}}\exp (-{\rm{i}}\omega t\mathrm{).}$$

**Figure 8:**
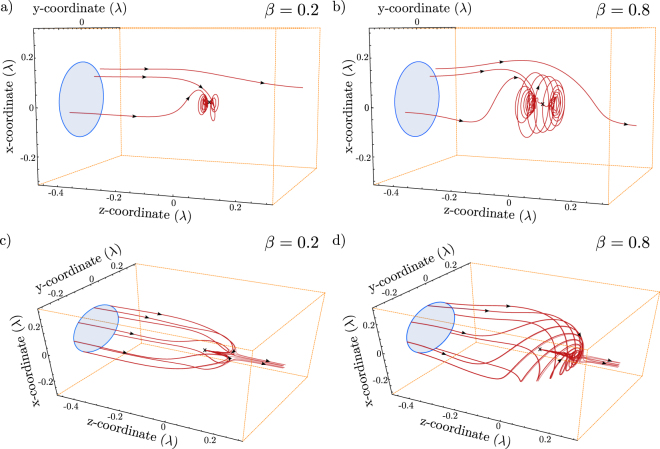
The absorption cross sections for a circular dipole. All incoming aperture shapes are circular. The dipole is located at the origin and marked with an **x**. The size of the absorption cross section is symmetric around *β* = 0.5. (**a**) Shows the energy flow for *β* = 0.2 for selected points inside and outside the aperture. The same points are followed in b) for the case of *β* = 0.8. The size of the cross section is the same. (**c**) and (**d**) show the energy flow for points slightly inside and outside of the aperture. Although the aperture is the same, the energy flow differs in the optical near-field. These pictures correspond to the calculations in ref.^31^, but show the energy flow *into* the dipole.

Notice the common phase shift of $\pi \mathrm{/2}$ between the incident wave and the reaction of the driven oscillator. The Eqns. 24 and 25 correspond to the Eqns. 1a and 16 in section 2. Based on this modification the same investigations as for the Hertzian dipole are performed. Figure 8 shows the apertures as well as some streamlines for the circular dipole case and different loss parameters. The way the energy travels into the dipole corresponds to the energy flow of a circular dipole emitter itself (without the incident beam) as investigated in Ref.^31^. One has to notice, that the direction of the energy flow in our case is towards the dipole, while the energy flow of a radiating dipole is logically outwards. This is due to the interference terms which direct the energy into the dipole. If the plots in Fig. 8a,b are compared, it is nicely visible that the behavior of the energy flow is more dominated by the scattered part for $\beta  > 0.5$. If one follows the solution which bypasses the dipole it occurs that the solution first spins around the *z*-axis until it changes the direction and gets “sucked” up into the dipole. This seems somehow counter intuitive, but simply results from the fact that both fields interfer in the proximity of the emitter. Figure 8c,d shows the boundary solutions for the two cases plotted in Fig. 8a,b.

**Figure 9:**
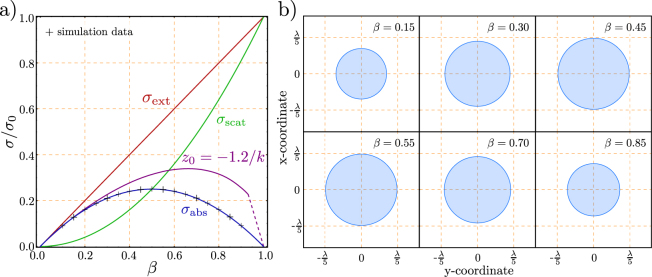
(**a**) The size dependence of a absorption aperture corresponds exactly the case for the Hertzian dipole (see Fig. 3a). The cases corresponding to the plots in b) Are highlighted with a bold+. (**b**) Apertures, far away from the dipolar emitter for different values of the loss parameter $\beta$. The determined values in a) correspond to calculations for different apertures. Unlike for the Hertzian dipole, we observe a shape symmetry against the value of $\beta =0.5$, although the energy flow is different, as shown in Fig. 8.

The pair of loss values (*β*) chosen for the plots in Fig. 8 are symmetric to 0.5. This can be seen in Fig. 3a/ Fig. 9a, and implies that the amount of energy absorbed by the dipole is equivalent in both cases. As shown in Fig. 9a the simulation data satisfies the theoretical forecast (which is exactly the same as for the linear case) for a circular excitation as well. But if the results are compared to the results of a Hertzian dipole it occurs that there is a shape symmetry against a loss value of 0.5 (Fig. 9b). This is more intuitive than for the Hertzian dipole case if one has the symmetric theory plot in mind (c.f. Fig. 3a). Moreover, it occurs that the shape of the absorption cross section stays perfectly round as visible in Fig. 9b. When the energy flow of a symmetric pair of loss values is investigated, it appears that the way the energy takes differs drastically for both cases (see also Fig. 8).

When the situations for the Hertzian and the circular dipole are compared, it is evident that both mathematical descriptions nicely yield a result which satisfies the underlying theory, independent of the incident wave polarization. Whereas the energy flow for the different cases differs totally, it always fulfills the boundary conditions that the “total area” of the absorption cross section yields to a corresponding result. Thus, it might be questionable how meaningful the energy flow itself is, since the definition of the Poynting vector is not unique. But the results of the simulation provided in the current paper may give an intuitive picture how light absorption occurs.

For the Hertzian dipole, it is determined, that the effective far-field aperture is altered in the optical near-field. This seems to be equivalently the case for the circular dipole. An example is depicted in Fig. 9a. These apertures are relatively easy to determine, since their shape is always circular, such that the derivation of one point on the boundary of the aperture is sufficient to calculate the area ($A=\pi {r}^{2}$).

## Conclusions

The presented results describe the situation regarding the power flow for a plane wave which interacts with a dipole emitter. They are fully equivalent to short antennas in the RF- or MW-range. The emitter can be a circular or a linear point-size dipole. To determine the absorption cross-section, the streamlines of the Poynting vector are followed, and result in an effective aperture, which matches the analytic derivations. The presented calculations are derived mostly equivalent to a situation with a focused light beam^18^ – the only difference is the definition of the incident field. Unlike there, the plane wave does not experience a Gouy phase shift and no reflection of light occurs with a plane wave. The later case corresponds more to a real measurement scenario than the studied case with a plane wave.

For a point-like Hertzian dipole, the apertures are not circular symmetric. The lateral area of the absorption cross-section is fully equivalent in both cases. Whereas for the linear case, the effective aperture changes its shape with the loss parameter, $\beta$, the circular dipole always has a circular shape.

In both cases, the apertures are changed in the optical near-field. This might not necessarily be interesting for far-field experiments with focused light. Also, extinction experiments in the optical near-field^2,32,33^ will be likely dominated by other effects, such as the emission of sub-wavelength apertures. Experiments with nano-particles or plasmonic structures will likely explore the described effects. For the nano-optical calculation and design of efficient absorbing structures, the presented results are of relevance. One example are optical wave-guides in which a single emitter can be placed^34^, such that the entire optical field interacts with high (ideally unity) probability with an emitter. Such calculations will allow to implement devices which are suitable for an efficient photon-photon interaction, which is required in many quantum optical proposals and is commonly known as a quantum phase gate.

The described math is presently extended to the case of multiple dipolar emitters, which might “cloak” each other by their presence. This yields situations comparable to Ref.^35^. Furthermore, we extend our approach to the case of a real optical focus, as described earlier^36,37^. This would correspond to a graphical representation of the case described by Ref.^18^.
